# Evaluation of Closed System Transfer Devices in Preventing Chemotherapy Agents Contamination During Compounding Process—A Single and Comparative Study in China

**DOI:** 10.3389/fpubh.2022.827835

**Published:** 2022-04-18

**Authors:** YiWen Tang, XiaoTian Che, Yao Lei Wang, Xin Ye, Wan Li Cao, Yi Wang

**Affiliations:** Department of Pharmacy, Huashan Hospital North, Fudan University, Shanghai, China

**Keywords:** CSTD, pharmacy, chemotherapy agents, closed system transfer devices, occupational protection

## Abstract

**Aim:**

We performed a comparative study to investigate the efficacy of closed system transfer devices (CSTDs) on the safe handling of injectable hazardous drugs (HDs).

**Methods:**

The exposure assessments of cyclophosphamide and cytarabine were performed under traditional or CSTDs. For preparation activity, chemotherapy contamination samples on protective equipment (such as gloves and masks) were collected. The contamination analysis was performed by liquid chromatography with tandem mass spectrometry (LC-MS/MS). A 6-item form was distributed monthly (form M1–M6, total 6 months) to assess the pharmacists' experience on ergonomics, encumbrance, and safety impression.

**Results:**

Totally, 96 wiping samples were collected throughout the study. The numbers of contaminated cyclophosphamide samples reduced under CSTD were −37.8, −41.6, −67.7, −47.3, and −22.9% and cytarabine were −12.3, −12.1, −20.6, −69.6, and −56.7% for left countertop, right countertop, medial glass, air-intake vent and door handle, as compared to traditional devices. The reduction was similar to pharmacist devices, i.e., −48.2 and −50.0% for masks and gloves cyclophosphamide contamination, −18.0 and −42.4% for cytarabine. This novel system could improve contamination on dispensing table, transfer container, and dispensing basket by −16.6, −6.0, and −22.3% for cyclophosphamide and −28.5, −22.5, and −46.2% for cytarabine. A high level of satisfaction was consistently associated with ergonomics for CSTD during the compounding process. Meanwhile, a slightly decreased satisfaction on ergonomics, encumbrance, and safety impression was observed for the traditional system between M2 and M3.

**Conclusion:**

Closed system transfer devices are offering progressively more effective alternatives to traditional ones and consequently decrease chemotherapy exposure risk on isolator surfaces.

## Introduction

Since long-term occupational exposure to chemotherapeutic agents in the pharmacy intravenous admixture service (PIVAS) was associated with serious health risks for compounding personnel, and furthermore, the health hazards were increased with the volume and frequency of exposure (1, 2). It was well known that exposure to certain antineoplastic drugs was hazardous to healthcare providers even at a very low exposure level, and currently, it was regarded as a crucial issue to limit the hazards with the protective equipment for healthcare providers who were exposed to chemotherapeutic agents (3). A previous study indicated that healthcare providers under the equipment with gloves, gowns, and goggles cautiously were less likely to be exposed to hazardous drugs (HDs) during compounding (4).

Nowadays, many health institutions, such as Occupational Safety and Health Administration (OSHA) or National Institute for Occupational Safety & Health (NIOSH), had realized the exposition hazards to chemotherapeutic agents and published guidelines to diminish professional risks, such as adverse reproductive reactions (including infertility and congenital malformations), skin rashes, and leukemia (5–7). As for the NIOSH alert publication, the application of closed system transfer devices (CSTD) for the preparation of chemotherapeutic agents has been increasing in hospitals (8). The application of CSTD is a drug transfer device that mechanically prevents the transfer of environmental contamination into the system and the leakage of dangerous drugs or vapor concentrates out of the system. Meanwhile, a national pharmacy practice survey supported by the American Society of Health-System Pharmacists (ASHP) found that about 41% of hospitals currently used CSTD to prevent HDs in an airtight and leak-proof manner (9). A current report documented that the CSTD could furthest diminish the potential exposure hazards to aerosols generated from chemotherapeutic agents and reduce the surface drug contamination (10).

Although the introduction of biological safety cabinet (BSC) had brought a reduction in exposure chemotherapeutic agents, which was recommended by current guidelines, preparation of anticancer drugs under conditions of BSC is still limited in China. Currently, the application advantages of CSTDs are recognized as effective prevention or reduction of HD exposures for health providers and pharmacists working in PIVAS. As a new protective measure, little information is available due to the production technology procurement and other reasons, limiting the development of CSTD in China. With the increasing number of CSTD, it is necessary to establish a process to determine various CSTDs available in the market. To investigate current conditions of CSTD in PIVAS, we performed a study to test the contamination of chemotherapeutic agents in working surfaces, gloves, gowns, and goggles followed the methodology outlined by the 2015 proposed NIOSH protocol.

## Materials

This study was conducted to evaluate the contamination in the chemotherapy agent compounding unit located in a teaching hospital of the university from January 2020 to January 2021. This is a tertiary hospital with 800 beds serving more than 25,000 people in Shanghai, China, and includes a comprehensive cancer center. The hospital PIVAS constantly provides dispensary, clinical and aseptic manufacturing services to the hospital cancer centers with a purpose-built aseptic suite and two pharmaceutical bio-safety cabinets dedicated to the preparation of chemotherapy agents according to USP 800.

The exposure assessments of cyclophosphamide and cytarabine were performed in personal protective equipment and different compounding areas in PIVAS. For preparation activity, contamination samples on the environment and protective equipment (such as gloves and masks) were handled and collected. The details of chemotherapy agent preparation activities (e.g., whether a spill occurred) and administration method were also recorded.

### Description of Chemotherapy Compounding Unit

The pharmaceutical team staffed with 8 pharmacists and 6 pharmacy technicians completed the preparations of 4,500 agents in the chemotherapy compounding unit annually. This isolated unit was designed according to International Organization for Standardization (ISO)-controlled atmosphere area equipped with biological safety cabinet (BSC-IIA2), which was filtered with High Efficiency Particulate Air filter (HEPA filter) with 70 kPa over-pressure toward the outside. Two pharmacists were engaged to chemotherapy agent compounding and wore disposable polychloroprene gloves and a disposable polypropylene gown, which had long sleeves and closed fronts throughout the procedure.

The BSC surfaces were cleaned thoroughly with 75% ethanol solution before the workday began. At the end of the work shift, the working surfaces were disinfected again and deep cleaning of the room floor and walls was conducted with chlorine-containing disinfectants.

### Study Design

**Figure 1:**
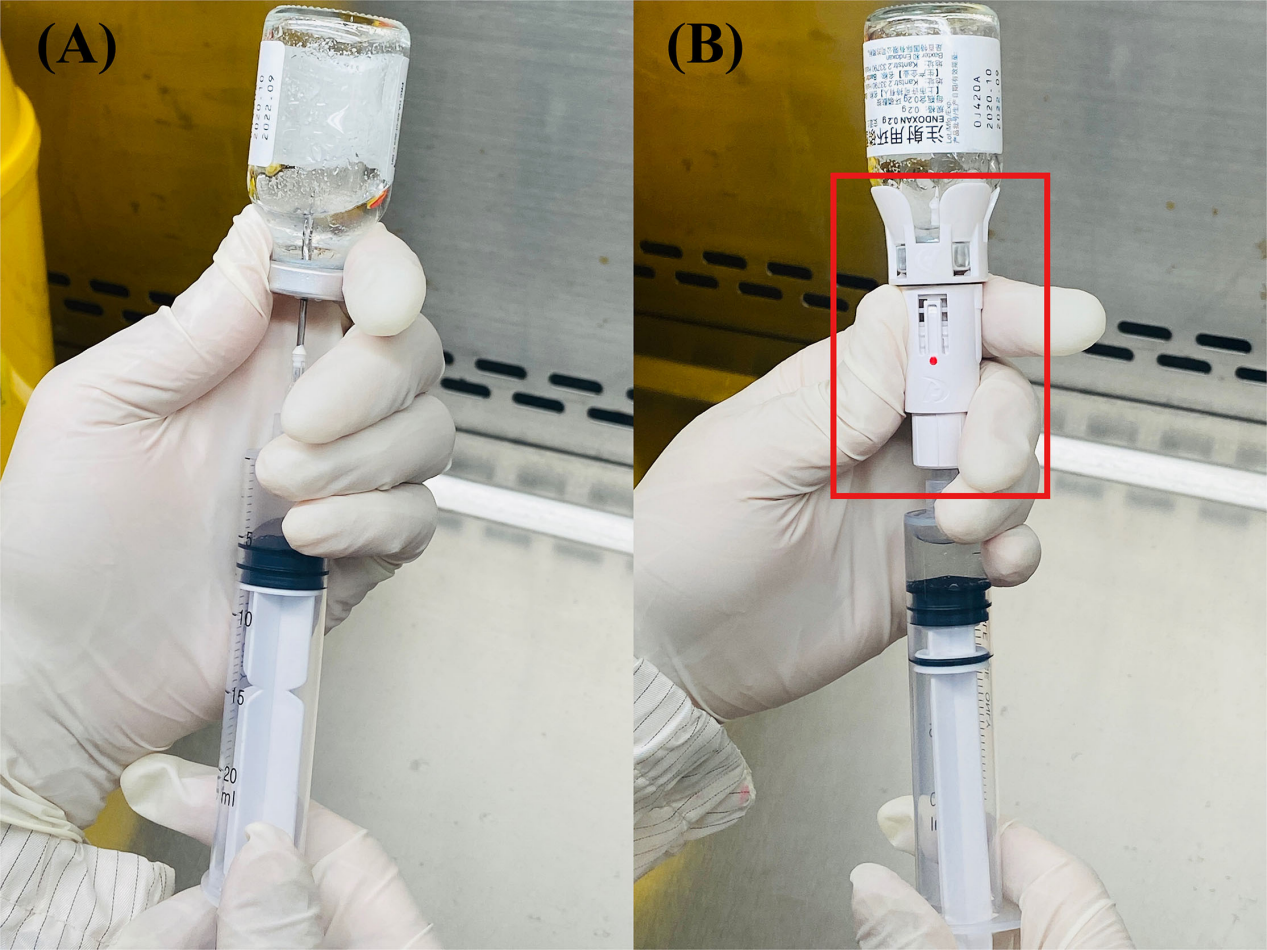
The structure of these two systems: traditional **(A)** and closed system transfer devices (CSTD) **(B)** From Shinva Ande Healthcare Apparatus Co., Ltd.

Our study was designed to evaluate the effectiveness of the CSTD system in reducing exposures of HDs from a stainless-steel surface of BSC. We collected two samples for each chemotherapy agents' measurement from the traditional and CSTD systems in the same chamber. The first sample was obtained prior to the mixture, and the second sample was obtained after compounding completion. All tested samples were collected by the pharmacists in PIVAS. The chemotherapy compounding process was completed through traditional needle syringe technique or CSTD systems, as shown in Figure 1. CSTD is mainly composed of a vial airtight access device, enclosed syringe safety device, enclosed baggage/line access device, and so on. All components in CSTD are sealed with resealing membranes. When components are joined together, the two membranes are pressed together and then pierced by the steel needle. The elastomeric double-membrane technology can ensure that there is no liquid medicine leakage of each component in the assembly and separation state. Therefore, the whole CSTD mechanically prohibits the transfer of environmental contaminants into the system and the escape of the HD or vapor concentrations outside the system.

**Figure 2:**
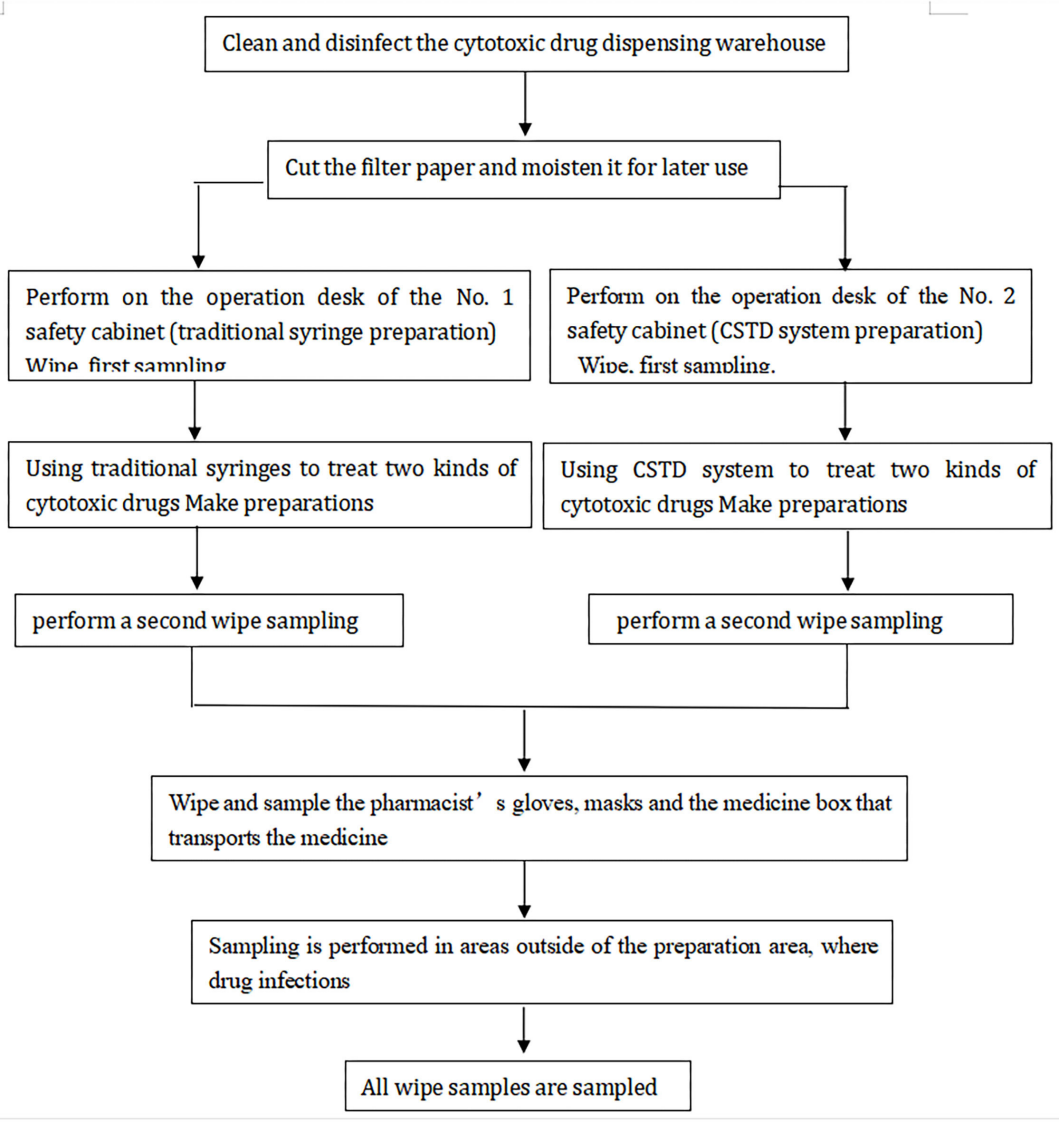
The study process for contamination prevention of two systems.

Surface contamination reductions of chemotherapy medications in traditional and CSTD units were compared to assess the effectiveness of the CSTD system. As for the traditional group, all chemotherapy agents compounding was completed with standard medical devices. For the CSTD group, the chemotherapy compounding was performed under personal protective equipment, which covered the whole process of injection preparation and administration (Figure 2).

### Sample Collection

The wiping sampling method allowed the verification of possible drug dispersion on the surfaces. Before the drug is flushed, we used 75% ethanol solution to thoroughly clean up the platform decently. During compound preparation, we took samples of various handles and pharmacist-used protective equipment (such as gloves and masks) in the active work area. Wipe samplings were conducted before cleaning and after preparation in both traditional and CSTD units. The sampled areas included left and right countertops of the operating area of the two BSCs, the inner side of the glass window of the BSC, and the front drain grooves. Each set of samples was taken before the isolator was cleaned at the end of the work session using a fresh 2 × 2 cm^2^ filler paper saturated by 100 μl prepared solution [Acetonitrile-0.1% formic acid aqueous solution (20:80)] for each surface. The area wiped from each location was 10 × 10 cm^2^ from two different directions, from up to down and from left to right in accordance with validated protocols. The collected samples did not touch any other surface and new pairs of gloves were provided to avoid potential contamination. The wipe papers were stored at 4°C prior to analysis. The details of chemotherapy agent preparation activities (e.g., prepared, checked, and whether a spill occurred) and administration method were also recorded.

### Contamination Samples Analysis

**Figure 3:**
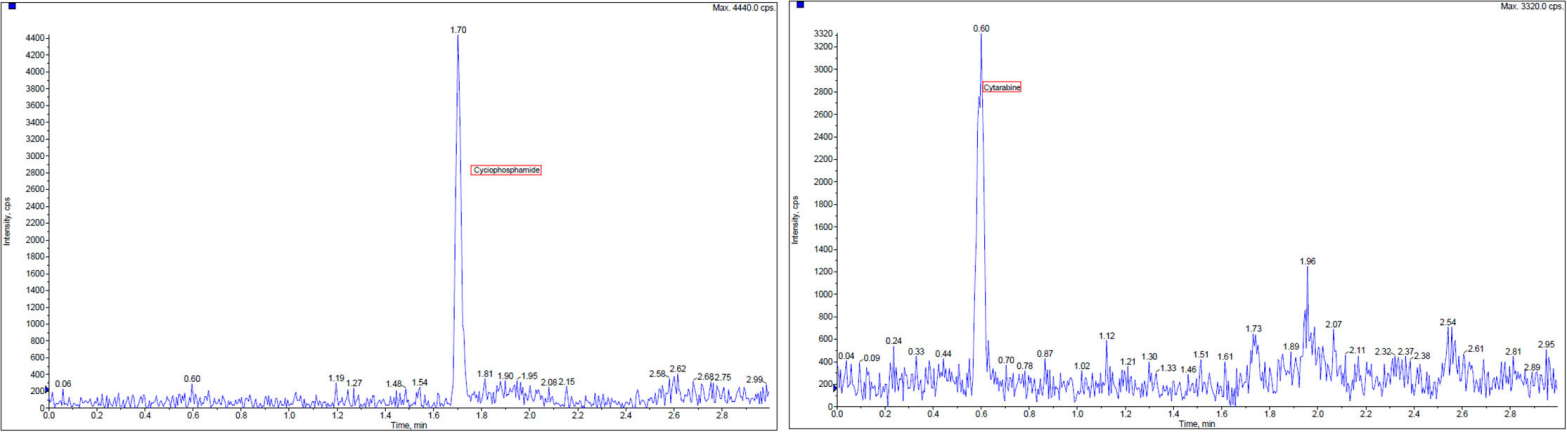
High performance liquid chromatography-mass spectrometry (HPLC-MS) chromatogram of extracted wipe samples containing two chemotherapy agents, cyclophosphamide and cytarabine, and verapamil as internal standards.

Cytarabine, cyclophosphamide, and verapamil (internal standards), all >98% purity, were obtained from Macklin Biochemical (Shanghai, China). Sample collection and preparation were conducted as the following process. A size of the filter paper (2 cm × 2 cm) was placed into 1.5 ml centrifuge tube. A volume of 950 μl acetonitrile and 0.1% formic acid (20:80) was added and after that 50 μl of internal standards verapamil, 10 ng/ml vortex was mixed for at least 15 s. The fully adsorption dissolving filter paper was taken out and the residual solution was centrifuged at 4°C and 11,000 r/min for 10 min. Then, transfer supernatant fluid of 300 μl was injected into LC-MS/MS for analysis. The limit of quantification (LOQ) was determined as the lowest concentration of the calibration curve, which met the following acceptance criteria was 0.5 ng/ml. The regression coefficients *r* were all >0.99, indicating good linearity. Blank samples were spiked at seven different concentrations of cyclophosphamide and cytarabine (10, 20, 100, 200, 1,000, 2,000, 4,500, and 5,000 ng/ml in the final extract) with a volume of 50 μl and carried out in accordance with the process of sample collection and preparation. A combination solution of 900 μl acetonitrile and 0.1% formic acid (20:80) and 50 μl internal standards verapamil 10 ng/ml was added. Calibration curves were constructed by plotting the concentrations on the X-axis vs. the chromatographic peak area ratio of ionic compounds internals standards on the Y-axis. Linear regression analyses were performed using the calibration curve data. The results of high performance liquid chromatography-mass spectrometry (HPLC-MS) profile for contamination wiping samples detection are reported in Figure 3.

### Estimation of the Usability of Devices by Pharmacists

A 6-item form was distributed monthly (form M1–M6, total 6 months) to assess the pharmacists' experience on ergonomics, encumbrance, safety impression and to establish a direct comparison between CSTD and traditional compounding process (Table 1). Each evaluation item was assigned a score from 0 to 5 points (1 = very bad, 2 = bad, 3 = neutral, 4 = good, and 5 = very good) with a total score of 30.

**Table 1:** Evaluation items on ergonomics, encumbrance, and safety impression.

**6-item Form evaluation for compounding devices**
**Ergonomics**
1) Connecting protector to vials
2) Connecting injector to syringes and protector
3) Injecting liquids into vials and containers
4) Withdrawing solution from vials and infusion bags
**Encumbrance**
**Safety impression in compounding process**

### Statistical Analysis

This trial was established to evaluate the impact of the CSTD system on exposure reduction of HDs of BSC. Based on the results of the previous clinical study, we supposed a difference of contamination variation between two compounding systems in prespecified analysis. Therefore, to preserve a one-sided type I error of 5% and adequate power, we selected a sample size of 84 samples to demonstrate a non-inferiority of different systems on contamination changes. Assuming subsequent losses to 15%, a whole study sample with 96 was required.

The reduction of contamination exposure levels of cyclophosphamide and cytarabine was compared between traditional and CSTD systems. The comparison general satisfaction on ergonomics, encumbrance, and safety impression of pharmacists were performed during the compounding process. The descriptive statistical results of continuous variables were expressed as means ± standard deviations (SD) and were compared using two-tailed Student's *t-*test between groups. Categorical data were presented as percentages. The chi-squared test was performed to compare the correlation of categorical variables. Statistical analysis was conducted using SPSS (IBM SPSS Statistics 22.0) and Prism 5 (GrandPad Software). The value of *p* < 0.05 was considered to be statistically significant.

## Results

### Contamination Wiping Samples Detection

Totally, 96 wiping samples were collected throughout the study, and wipe sample sites are presented in Table 2. The exposure reduction efficiency was calculated by contamination per square centimeter and all results were underestimated.

**Table 2:** Summaries of wiping samples for traditional and closed system transfer device (CSTD) systems.

**Area (cm^**2**^)**	**No. of detectable samples (Traditional)**	**No. of detectable samples** **(CSTD)**
BSC		
Countertop, Left (100 cm^2^)	5	5
Countertop, Right (100 cm^2^)	5	5
Medial glass (100 cm^2^)	5	5
Air-intake vent (100 cm^2^)	5	5
Door handle (48 cm^2^)	5	5
Pharmacist devices		
Masks (16 cm^2^)	4	4
Gloves (4 cm^2^)	4	4
Drug administration		
Dispensing table (100 cm^2^)	5	5
Transfer container (100 cm^2^)	5	5
Dispensing basket (100 cm^2^)	5	5

*BSC, biosafety cabinet; cm^2^, square centimeter*.

### Contamination Description of two Systems

There was almost no chemotherapy agent residual prior to compounding. After compounding, the levels of the chemotherapy agent contamination of cyclophosphamide and cytarabine were recovered from wipe samples during traditional and CSTD phases, which are shown in Table 2.

In the BSC area, CSTD could significantly reduce cyclophosphamide contamination exposure as compared to the traditional system. Similarly, cytarabine contamination in the location of the air-intake vent and door handle could be significantly reduced under the condition of CSTD. Meanwhile, no significant decrease in cytarabine exposure contamination was found between the two systems. As for protective equipment, the chemotherapy agent contamination was significantly lower under the CSTD, whatever the localization. During the administration process, CSTD could significantly decrease cytarabine contamination.

**Figure 4:**
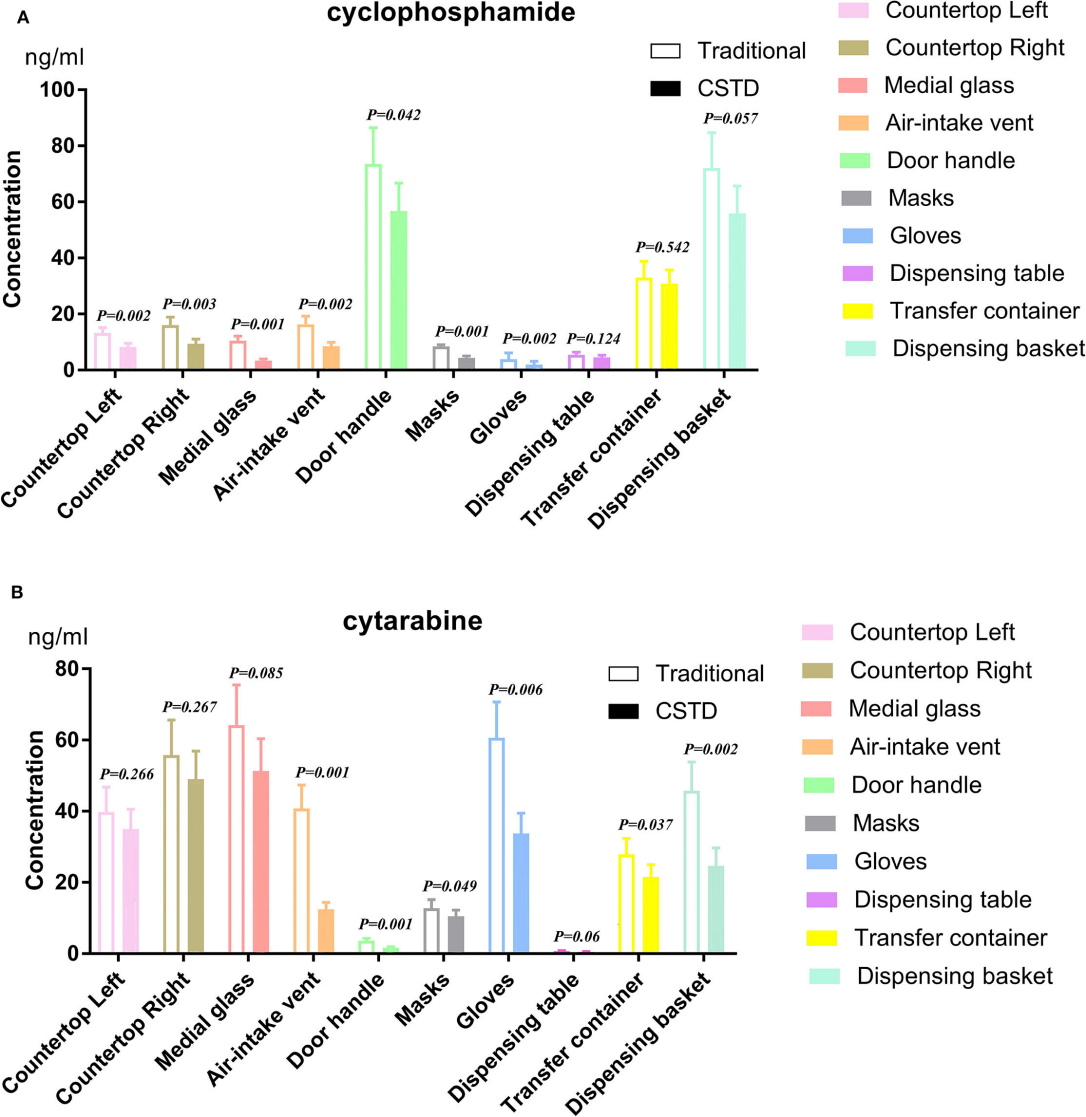
Cyclophosphamide and cytarabine on isolator surfaces and preparation with traditional and CSTD intervention. *p* < 0.05 was regarded as a significant difference. **(A)** cyclophosphamide, **(B)** cytarabine.

As depicted in Figure 4, the percentage decrease values under CSTD are −37.8, −41.6, −67.7, −47.3, and −22.9% of cyclophosphamide samples and −12.3, −12.1, −20.6, −69.6, and −56.7% of cytarabine samples for left countertop, right countertop, medial glass, air-intake vent, and door handle when compared to traditional units. Similar trends were observed in pharmacist-used protective equipment: −48.2 and −50.0% for cyclophosphamide and −18.0 and −42.4% for cytarabine. Likewise, cyclophosphamide contamination percentage was found with −16.6, −6.0, and −22.3% for dispensing table, transfer container, and dispensing basket under CSTD conditions, respectively. The percentages of contaminated cytarabine samples are −28.5, −22.5, and −46.2%, respectively, with the traditional devices, as shown in Figure 4.

### Usability Evaluation of Devices by Pharmacists

Totally, 14 pharmacists completed the 6-item form each month during the study. At the beginning of the study, the general satisfaction on ergonomics, encumbrance, safety impression of pharmacists was moderate (medium) for both CSTD and traditional systems.

**Figure 5:**
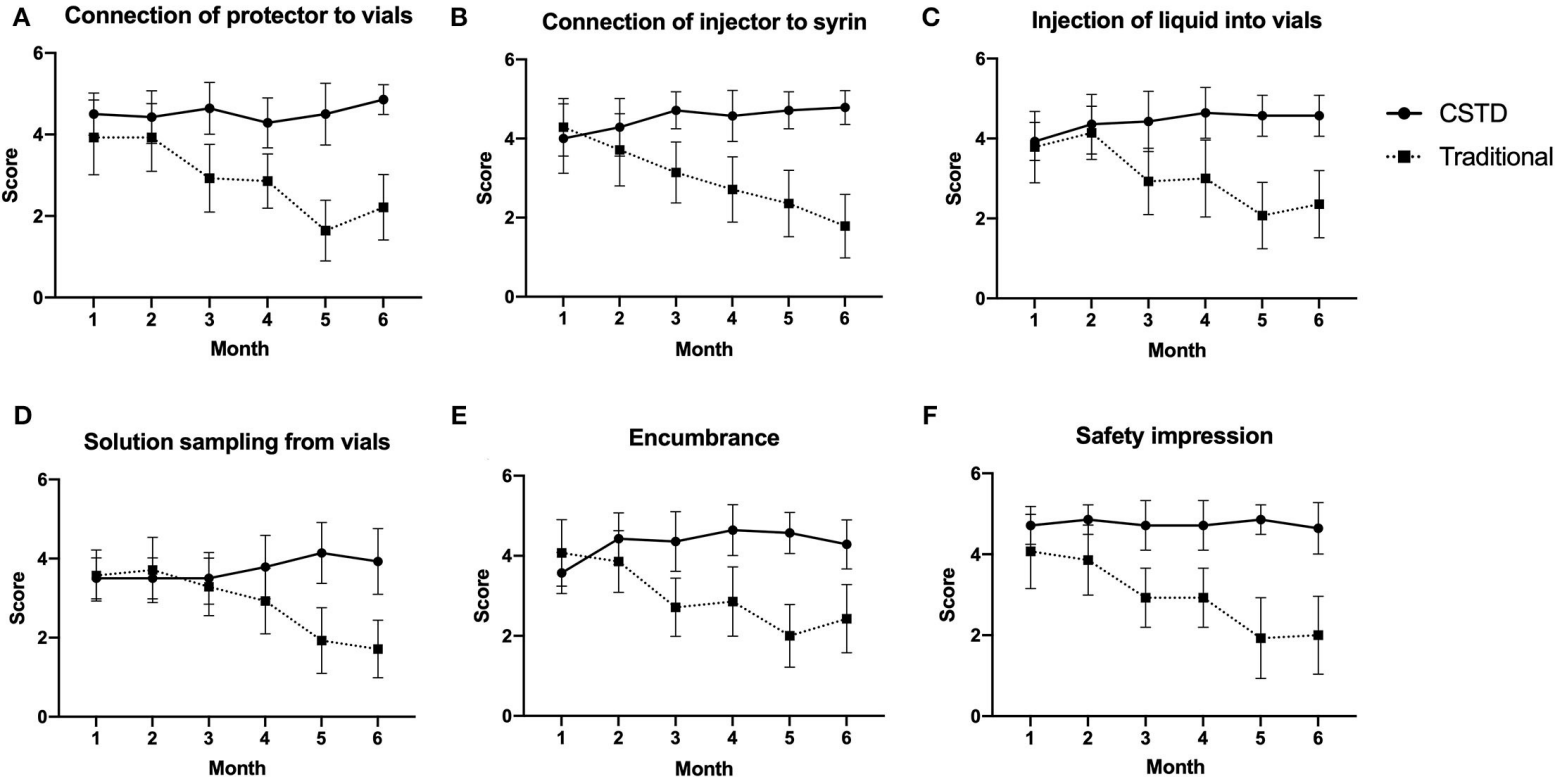
The satisfaction comparison of ergonomics, encumbrance, and safety impression. **(A)** Connection of protector to vials, **(B)** connection of injector to syrin, **(C)** injection of liquid into vials, **(D)** solution sampling from vials, **(E)** encumbrance, **(F)** safety impression.

As depicted in Figure 5, the satisfaction on encumbrance and safety impression for the CSTD system is good from the start and remained constant throughout the whole study. A high level of satisfaction was consistently associated with ergonomics for CSTD during the compounding process. Meanwhile, a slightly decreased satisfaction on ergonomics, encumbrance, and safety impression is observed for the traditional system between M2 and M3, as shown in Figure 5.

## Discussion

Reduction in surface contamination for compounding areas and pharmacist devices had raised major concern for many years in PIVAS. Our study indicated that adjacent preparation areas in BSCs, pharmacist device, and drug administration locations might be associated with decreased contamination during the chemotherapy agent compounding process when CSTD systems were used. Moreover, a superior experience on ergonomics, encumbrance, and safety impression was found during CSTD system application as compared to the traditional system. The results showed that CSTD could greatly reduce the pollution of cytotoxic on the BSC and the environment and reduce the waste of medical resources.

This is the first direct comparative study to investigate the degree of contamination by chemotherapeutic agents in the working environment or to healthcare providers who applied the traditional or CSTD handling practice in China. Our results demonstrated that CSTDs could significantly reduce chemotherapy agent contamination, including cyclophosphamide and cytarabine, on isolate surfaces as compared to traditional systems. Currently, CSTDs are not widely used and accessible to chemotherapy agents compounding in hospital settings, and this study might provide clinical evidence for the superiority of CSTDs.

The preliminary results indicated that contamination with cyclophosphamide and cytarabine was primarily identified in most areas of the aseptic dispensary unit and pharmacist device. Surface contamination might be caused by several factors in the clinical settings, including the original pollution residue of the medicine bottle, the indirect pollution caused by the contaminated gloves, and the improper operation (9). One common source of contamination originated from aerosol formation due to the pressure inside the drug vial (11, 12). Previous literature also demonstrated residual chemotherapy agent contamination on the exterior of vials received from pharmaceutical manufacturers (13, 14). High concentration level of chemotherapy agent contamination was identified on the countertop location inside the BSC, personal protective equipment, such as masks, gloves, and drug administration areas. It could be explained that cyclophosphamide and cytarabine spillage are easily exposed in those areas during the compounding process. Our results were consistent with the previously reported articles, which had conducted the residual cyclophosphamide contamination concentration assessment in a similar method (10, 15, 16). It was reported that the inside areas of aseptic dispensary unit were associated with a high possibility of contamination and the countertop inside the BSC was considered as a higher possibility of contamination location. The phenomenon could be explained by some spillage occurrences during the compounding process.

Many cautionary documents demonstrated that the lymphocyte DNA damage was 5–7-fold more common in healthcare providers who handle chemotherapy agents than the normal (17–19). Although BSC could prevent most environmental contamination that is mainly caused by hazardous spills and aerosol, residual contamination still existed which might bring long-term hazards. Many studies reported that healthcare providers handling with HDs who suffered a miscarriage, fertility, and birth defects were detected measurable urine chemotherapy agent concentrations (2, 9). CSTDs could limit the chemotherapy agent spillage and provide a safer environment, mainly attributing to the high efficiency of particulate air (HEPA) filter supply and air extraction from the BSC (20, 21).

Our results demonstrated a significant reduction in the cyclophosphamide and cytarabine contamination levels in most detected areas after implementing CSTD methods during the preparation process. As shown in Table 2 of our study, CSTDs could significantly reduce average values of cyclophosphamide and cytarabine contamination concentration. Especially for masks and gloves directly exposed to chemotherapy agents, CSTDs could reduce the percentage of contamination by 48.2 and 50.0%, respectively, based on the average contamination of the traditional method, in agreement with similar studies performed earlier (22, 23). The present study was consistent with this conclusion. The probable explanation might be that CSTDs could mechanically prevent the contamination of environmental impact on the system and the leakage or vapor concentrates of HDs out of the system.

As for pharmacists' observations, general satisfaction with the CSTDs remained constantly good throughout this study, while it was good from the beginning and continuously decreased over the whole study period for traditional systems. The application of CSTDs was now hailed as an alternative safety measure due to the full awareness of relatively high risks of chemotherapy agent handled in clinical settings. Our teams were trained to operate this novel device correctly during the compounding process to avoid technical problems caused by human factors. The results indicated that CSTDs could improve pharmacists' satisfaction on ergonomics, encumbrance, and safety impressions. The probable reason might be that the safety of CSTDs helped pharmacists to modify perception, which not only increased the degree of satisfaction but also improved the effectiveness of the operation.

## Conclusion

Currently, the topic of measures taken to protect handlers from occupational exposure to chemotherapy has always been controversial. The proper use of a CSTD may significantly decrease contamination by these drugs and consequently decrease exposure risk on workplace surfaces and personal protective equipment as compared to traditional compounding devices.

### Limitations

We acknowledge several limitations in our work. First, owing to the nature of this observational study, we have some relevant limitations, such as selection bias or the single-center observation, which might have an impact on research quality. Second, the study included a limited number of samples obtained during this preliminary study. Hence, large sample size is needed in further study. Third, the absent determination of contamination on the drug vials might influence the statistical summary of the accuracy and precision, and further study and statistical analyses are needed to investigate the containment effectiveness of the two systems in a controlled setting.

## Data Availability Statement

The original contributions presented in the study are included in the article/Supplementary Material, further inquiries can be directed to the corresponding author.

## Ethics Statement

Ethical review and approval was not required for the study of human participants in accordance with the local legislation and institutional requirements. Written informed consent from the patients/ participants was not required to participate in this study in accordance with the national legislation and the institutional requirements.

## Author Contributions

YT: conceptualization, methodology, and writing—original draft. YW: writing—review & editing. WC and XY: supervision. YLW: validation. XC: acquisition of data, analysis, and interpretation of data. All authors contributed to the article and approved the submitted version.

## Conflict of Interest

The authors declare that the research was conducted in the absence of any commercial or financial relationships that could be construed as a potential conflict of interest.

## Publisher's Note

All claims expressed in this article are solely those of the authors and do not necessarily represent those of their affiliated organizations, or those of the publisher, the editors and the reviewers. Any product that may be evaluated in this article, or claim that may be made by its manufacturer, is not guaranteed or endorsed by the publisher.
